# Lipopolysaccharide Induces Pro-Inflammatory Cytokines and MMP Production via TLR4 in Nasal Polyp-Derived Fibroblast and Organ Culture

**DOI:** 10.1371/journal.pone.0090683

**Published:** 2014-11-12

**Authors:** Jung-Sun Cho, Ju-Hyung Kang, Ji-Young Um, In-Hye Han, Il-Ho Park, Heung-Man Lee

**Affiliations:** 1 Brain Korea 21 Plus for Biomedical Science, College of Medicine, Korea University, Seoul, South Korea; 2 Institute for Medical Devices Clinical Trial Center, Korea University, Guro Hospital, Seoul, South Korea; 3 Department of Otorhinolaryngology-Head and Neck Surgery, Korea University, Guro Hospital, Seoul, South Korea; University of Kansas School of Medicine, United States of America

## Abstract

Nasal polyposis is characterized by persistent inflammation and remodeling in sinonasal mucosa. Toll-like receptors (TLRs) play a role in the innate immune response to microbes in the sinonasal cavity. The aim of this study was to evaluate whether nasal polyp-derived fibroblasts (NPDFs) and organ-cultured nasal polyps can synthesize pro-inflammatory cytokines and matrix metalloproteinases (MMPs) after exposure to lipopolysaccharide (LPS), a TLR4 agonist. NPDFs and organ-cultured nasal polyps were isolated from nasal polyps of 8 patients and exposed to LPS. The mRNA and protein expression levels of TLRs, cytokines, and MMPs were determined using a gene expression microarray, real-time RT-PCR, western blot analysis, enzyme-linked immunosorbent assay, and immunofluorescence staining. The enzymatic activities of MMPs were analyzed using collagen or gelatin zymography. The protein expression level of MMP-1 increased in nasal polyp tissues compared to inferior turbinate tissues. LPS induced mRNA expression of *TLR4*, *IL-6*, *IL-8*, and *MMP-1* and activated MAPK signaling in NPDFs. LPS promoted the release of interleukin (IL)-6 through extracellular signal-related kinase (ERK) and IL-8 through ERK and c-Jun N-terminal kinases (JNK). Production of IL-6 and IL-8 was induced by PI3K/Akt signaling in LPS-stimulated NPDFs. LPS increased the transcript and protein expression levels of MMP-1 and induced collagenase activity of MMP-1 via ERK and p38, but did not induce gelatinase activity of MMP-2 and MMP-9. LPS from *Rhodobacter sphaeroides* (LPS-RS) inhibited the stimulatory effects of LPS in NPDFs as well as in organ culture of nasal polyp. LPS triggers immune response via TLR 4 and activates MAPK and PI3K/Akt signaling pathway, which is involved in remodeling of nasal polyps.

## Introduction

Chronic rhinosinusitis with nasal polyps (CRSwNP) is a form of sinonasal inflammatory disease characterized by persistent eosinophilic inflammation, edematous mucosa with nasal polyps, and thickened sinonasal secretions [1], [2]. Although it has been proposed that CRSwNP is fundamentally an inflammatory disease rather than an infection, it also has been hypothesized that microbes often present in the sinonasal cavity play a role in initiating or perpetuating mucosal inflammation [3], [4]. As the first site of contact between the host and outside environment, the sinonasal cavity plays a critical role in immunity.

Fibroblasts are major structural components of tissues, where they confer mechanical strength by providing a supporting framework for the extracellular matrix (ECM) and are also thought to be responsible for local recruitment of inflammatory cells owing to their ability to produce a variety of chemokines [5], [6]. Although fibroblasts play an important role as a source of biological mediators in initiating and amplifying inflammation, overproduction of these factors by fibroblasts may prevent resolution of this condition, leading to chronic inflammation [7]. Whether bacterial products, such as lipopolysaccharide (LPS), can directly elicit cytokine responses in fibroblasts remains controversial [8], [9].

Toll-like receptors (TLRs) are transmembrane receptors with an extracellular domain that interacts with a pathogen ligand and an intracellular domain that is involved in signaling [10]. Mammals express at least 10 TLRs that recognize specific pathogen molecules. Each of these TLRs is thought to play a role in the innate immune response to innocuous microbes in the sinonasal cavity as well as airborne bacterial, fungal, or viral pathogens; for example, TLR4 recognizes LPS from gram-negative bacteria. The mRNA for all 10 TLRs is expressed in the sinonasal mucosa, both in health and in sinus disease [11]. Although the mechanisms underlying persistent inflammation in CRS are unknown, innate immune processes may play a role.

Matrix metalloproteinases (MMPs) comprise a large family of proteolytic enzymes containing a zinc-binding catalytic domain and are involved in the degradation of ECM components. Their extracellular activities are regulated by tissue inhibitors of MMP (TIMP) [12]. Fibroblasts also secrete MMPs that may contribute to tissue destruction [13]. Expression levels of MMPs have been found to be elevated in nasal polyp tissues compared to control tissues and play important roles in the formation of nasal polyposis [14], [15]. However, the role of LPS-induced pro-inflammatory cytokines and MMPs in nasal polyp-derived fibroblasts (NPDFs) has not been reported.

We hypothesized that LPS exposure up-regulates not only pro-inflammatory cytokines, but also tissue remodeling via MMPs in patients with nasal polyposis. In this study, we evaluated whether NPDFs and organ-cultured nasal polyps can synthesize pro-inflammatory cytokines and MMPs following exposure to LPS.

## Materials and Methods

### Materials

LPS from *Pseudomonas aeruginosa* was obtained from Sigma (St. Louis, MO). InvivoGen (San Diego, CA) provided LPS isolated from the photosynthetic bacterium *Rhodobacter sphaeroides* (LPS-RS). Inhibitors of extracellular related kinase (ERK) (U0126), p38 (SB203580), c-Jun N-terminal kinase (JNK) (SP600125) and PI3K/Akt inhibitor (LY294002) were purchased from Calbiochem (Billerica, MA). The inhibitors were dissolved in dimethyl sulfoxide. Antibodies against phospho-ERK, phospho-p38, and phospho-JNK were purchased from Cell Signaling Technology (Danvers, MA). Antibodies against TLR4 (sc-10741), MMP-1 (sc-21731), MMP-2 (sc-10736), MMP-9 (sc-21733), GAPDH (sc-47724), and β –actin (sc-4778) were obtained from Santa Cruz Biotechnology (Santa Cruz, CA).

### Nasal polyp tissues and NPDF culture

Nasal polyp tissues were collected from 8 patients with chronic rhinosinusitis (CRS) and nasal polyp who were recruited from the Department of Otorhinolaryngology at the Korea University Medical Center. No patients had a history of allergy, asthma, or aspirin sensitivity, and they had not been treated with oral anti-allergic agents for at least 2 months. Written informed consent was obtained from each patient, and the study was approved by the Korea University Medical Center Institutional Review Board (KUGH12041). NPDFs were isolated from surgical tissues and purified according to our previous study [16]. Cells used for experiments were obtained from the fourth cell passage.

### Microarray analysis

NPDFs (5×10^6^ cells/mL) were exposed to LPS (10 µg/mL) for 12 h. Total RNA was isolated using Trizol reagent (Invitrogen, Carlsbad, CA). For control and test RNAs, synthesis of target cRNA probes and hybridization were performed using the Low RNA Input Linear Amplification kit (Agilent Technology, Santa Clara, CA). Hybridized images were scanned using a DNA microarray scanner and quantified using Feature Extraction Software (Agilent). All data normalization and selection of fold-change of the genes were performed using GeneSpringGX 7.3 (Agilent) in Table 2. The accession number of our microarray data in GEO (Gene Expression Omnibus) is GSE52505.

**Table 1:** Sequences of RT-PCR oligonucleotide primers.

Primer	Direction	Sequence	Size (bp)
**TLR4**	forward	5′- TGA GCA GTC GTG CTG GTA TC -3′	167
	reverse	5′- CAG GGC TTT TCT GAG TCG TC -3′	
**IL-6**	forward	5′- GGT ACA TCC TCG ACG GCA TCT -3′	81
	reverse	5′- GTG CCT CTT TGC TGC TTT CAC -3′	
**IL-8**	forward	5′- ATG ACT TCC AAG CTG GCC -3′	282
	reverse	5′- TCT TCA AAA ACT TCT CCA CAA CCC -3′	
**MMP-1**	forward	5′- CAG AGA TGA AGT CCG GTT TTT C -3′	75
	reverse	5′- GGG GTA TCC GTG TAG CAC AT -3′	
**MMP-2**	forward	5′- AGA TCT TCT TCT TCA AGG AAC CGT T -3′	224
	reverse	5′- GGC TGG TCA GTG GCT TGG GGT A -3′	
**MMP-9**	forward	5′- GCG GAG ATT GGG AAC CAG CTG TA -3′	208
	reverse	5′- GAC GCG CCT GTG TAC ACC CAC A -3′	
**GAPDH**	forward	5′- GTG GAT ATT GTT GCC ATC AAT GAC C -3′	271
	reverse	5′- GCC CCA GCC TTC TTC ATG GTG GT -3′	

**Table 2:** Genes significantly upregulated in LPS-induced NPDFs.

GeneSymbol	Genebank	Description	LPS/control ratio
**Upregulated**			
**TLR4**	NM_138554	toll-like receptor 4	4.170
**IL-1B**	NM_000576	interleukin 1, beta	3.487
**IL-6**	NM_000600	interleukin 6 (interferon, beta 2)	2.091
**IL-8**	NM_000584	interleukin 8	722.055
**IL-11**	NM_000641	interleukin 11	9.918
**IL-16**	NM_172217	interleukin 16 (lymphocyte chemoattractant factor)	2.189
**IL-17B**	NM_014443	interleukin 17B	3.621
**IL-32**	NM_001012631	interleukin 32	6.942
**IL-33**	NM_033439	interleukin 33	4.217
**MMP-1**	NM_002421	matrix metallopeptidase 1 (interstitial collagenase)	239.883
**MMP-3**	NM_002422	matrix metallopeptidase 3 (stromelysin 1, progelatinase)	132.091
**MMP-12**	NM_002426	matrix metallopeptidase 12 (macrophage elastase)	25.454
**MMP-14**	NM_004995	matrix metallopeptidase 14 (membrane-inserted)	22.300
**MMP-24**	NM_006690	matrix metallopeptidase 24 (membrane-inserted)	3.240
**MMP-28**	NM_001032278	matrix metallopeptidase 28	2.084
**TIMP-1**	NM_003254	TIMP metallopeptidase inhibitor 1	2.026

### Real-time RT-PCR

Total tissue RNA was extracted using NucleoSpin RNA II (Macherey-Nagel, Düren, Germany) according to the manufacturer's instructions. The RNA concentration was determined using the NanoDrop ND-2000 Spectrophotometer (Thermo Scientific, Wilmington, DE). Quality and integrity of total RNA was assessed on 1% formaldehyde-agarose gels. For real-time RT-PCR, 2 µg of total RNA in a 50 µl reverse transcriptase reaction mixture was reverse transcribed to cDNA by using the ReverTra Ace qPCR RT Kit (Toyobo, Osaka, Japan) following the manufacturer's protocols. Quantitative PCR was then carried out in a 7300 Real-Time PCR System (Applied Biosystems, Foster City, CA) using 2 µL of cDNA template, 80 nM of each primers, and 12.5 µL of Power SYBR Green PCR Master Mix (Applied Biosystems, Foster City, CA) in a total volume of 25 µL. The forward and reverse primers used for PCR are shown in Table 1. The cDNA were amplified with an initial denaturation step at 95°C for 10 min followed by 40–50 cycles of PCR with the following program: 95°C for 15 s, 58°C for 60 s, and 1 cycle of melting curve following cooling at 60°C for 60 s. To confirm amplification specificity, the PCR products for each primer pair were subjected to melting-curve analysis. Analysis of relative gene expression was conducted by evaluating quantitative RT-PCR data using the 2^(−ΔΔCt)^ method. Each experiment was repeated at least 3 times and *GAPDH* was used as an internal control.

### Western blot analysis

NPDFs were exposed to LPS with or without LPS-RS for 72 h. To examine the MAPK signaling pathway, NPDFs were previously exposed to LPS after pretreatment with MAPK inhibitors, including U0126, SB203580, and SP600125. Samples were lysed in PRO-PREP^™^ protein extraction solution (iNtRON Biotechnology, Seongnam, Korea). Lysates were separated by 10% SDS-PAGE and transferred to polyvinylidene fluoride (PVDF) membranes (Millipore Inc., Billerica, MA). Membranes were blocked with 5% skim milk solution and incubated with the following antibodies: TLR4, MMP-1, MMP-2, MMP-9, phospho-ERK, phospho-p38, phospho-JNK, and GAPDH. Blots were visualized using horseradish peroxidase-conjugated secondary antibodies and an enhanced chemiluminescence system (Pierce, Rockford, IL).

### Enzyme-linked immunosorbent assay (ELISA)

IL-6 and IL-8 concentrations in the culture medium were determined using ELISA (R&D systems, Minneapolis, MN). NPDFs were previously exposed to LPS for 72 h after pretreatment with MAPK inhibitors, including U0126, SB203580, and SP600125. Standards and samples were added and incubated at room temperature for 2 h. After 3 washes, IL6 or IL-8 conjugate was added to the wells for 2 h at room temperature. The reaction was stopped with a stop solution, and the product was quantified at 450 nm using a microplate reader (Bio-Rad).

### Immunofluorescence staining

For immunoflurorescent staining, 5-µm-thick sectioned tissues of human inferior turbinate, nasal polyp, and organ-cultured nasal polyp or NPDFs were fixed with 4% paraformaldehyde. Tissues and NPDFs were permeabilized with 0.2% Triton X-100 containing 1% bovine serum albumin (BSA) for 10 min, blocked with 5% BSA for 1 h at room temperature, and incubated overnight at 4°C with antibodies against TLR4, MMP-1, MMP-2, and MMP-9. NPDFs were then incubated with anti-mouse Alexa 488 (Invitrogen) or anti-rabbit Alexa 555 (Invitrogen) secondary antibodies. Finally, coverslips were counterstained with 4′,6-diamidino-2-phenylindole (DAPI). Images of each stained NPDF were captured and visualized using confocal laser scanning microscopy (LSM700, Zeiss, Oberkochen, Germany).

### Gelatin and collagen zymography

Aliquots of medium conditioned by cells were analyzed using gelatin zymography for MMP-2 and MMP-9 in 1 mg/mL gelatin-10% polyacrylamide gels or collagen zymography for MMP-1 in 0.4 mg/mL collagen-10% polyacrylamide gels. Following electrophoresis, the gels were washed twice with 2.5% Triton X-100 for 30 min while shaking to remove SDS and to renature the MMP-2, MMP-9, or MMP-1 in the gels. Renaturated gels were incubated in developing buffer containing 100 mM Tris–HCl, 5 mM CaCl_2_, 0.005% Brij-35, and 0.001% NaN_3_ (pH 8.0), overnight at 37°C. Gels were stained with 0.25% Coomassie brilliant blue G-250 (50% methanol, 10% acetic acid) and destained using destaining solution (50% methanol, 10% acetic acid). Proteinase activity was observed as cleared (unstained) regions. Finally, the gels were dried for 2 h using a gel dryer (Bio-Rad Labs).

### Ex vivo organ culture

Nasal polyp tissues were cut into 2 to 3 mm^3^ pieces under sterile conditions. Tissue fragments were washed 3 times with phosphate-buffered saline and then rinsed with culture medium. The culture medium was composed of 98% Dulbecco's minimum essential medium (Invitrogen), 2% heat-inactivated fetal bovine serum (Invitrogen), 100 U/mL penicillin (Invitrogen), 100 µg/mL streptomycin (Invitrogen), and 0.25 µg/mL fungizone. The rinsed tissue fragments were placed on pre-hydrated 10×10×1 mm gelatin sponge (Spongostan, Johnson & Johnson, San Angelo, TX) with the mucosa side facing up and the submucosa side facing down. Tissue fragments were placed into 6-well plates and filled with 1.5 mL of culture medium per well such that the mucosa was above the liquid phase. Nasal polyps were stimulated with LPS (20 µg/mL) with or without LPS-RS (20 µg/mL) for 72 h. The cultured nasal polyps were examined to detect protein expression levels of IL-6, IL-8, and MMP-1. The plates were placed in a humidified incubator and maintained at 37°C in 5% CO_2_.

### Statistical analysis

The results were obtained from at least 3 independent experiments. The statistical significance of the differences between control and experimental data was analyzed using the unpaired *t*-test or one-way analysis of variance (ANOVA) followed by Tukey's test (GraphPad Prism, version 5, GraphPad Software, San Diego, CA). Significance was established at the 95% confidence level. P-values <0.05 were accepted as statistically significant.

## Results

### MMP-1 protein expression levels are increased in nasal polyp tissues

**Figure 1:**
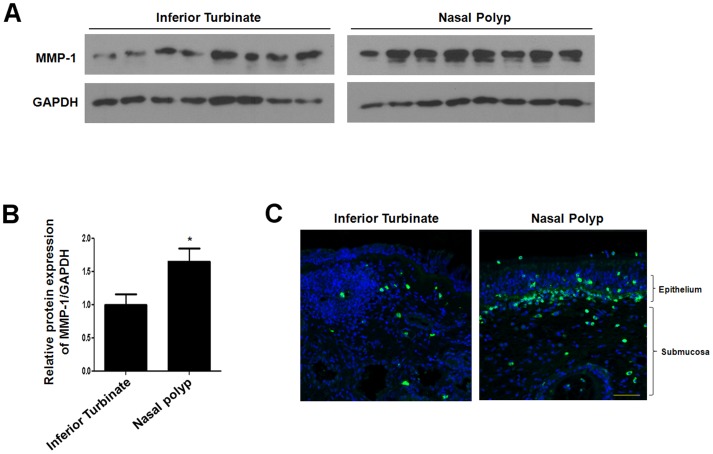
Expression of MMP-1 in inferior turbinate and nasal polyps. Protein expression level of MMP-1 based on western blot analysis (A) and density analysis (B). (C) The expression pattern and localization of MMP-1 based on immunofluorescence staining. Images were acquired and visualized using confocal z-stack laser scanning microscopy. Values are the mean ± SEM of independent samples. Asterisks (*) indicate statistically significant differences (P<0.05). Scale bar  = 50 µm.

In a recent study, MMP-1 expression was shown to be significantly increased in nasal polyps and CRS compared with normal nasal mucosa [15]. To examine MMP-1 protein expression, we compared MMP-1 expression level in inferior turbinate and nasal polyp tissues. Western blot analysis showed that MMP-1 protein expression levels increased in nasal polyp tissues compared to inferior turbinate tissues (Fig. 1A and 1B). MMP-1 protein was primarily localized at the basement membrane and expressed in the cytoplasm of fibroblasts and inflammatory cells of nasal polyp tissue (Fig. 1C). These results demonstrate that an increased protein expression level of MMP-1 is related to nasal polyposis.

### LPS alters gene expression profiles in NPDFs

To investigate the effect of LPS on gene expression levels in NPDFs, NPDFs were treated with LPS and examined using gene expression microarray analysis. Of the 34,127 genes represented in the Whole Human Genome Microarray Kit (4×44K), the genes showing significant differences greater than 2.0 fold between control and LPS groups were selected for further analysis. These genes were regarded as differentially expressed following LPS exposure compared to the control in NPDFs, and included 1297 up-regulated probes and 7462 down-regulated probes. Inflammation- and immune response-related genes up-regulated or down-regulated following LPS stimulation included those encoding TLRs, ILs, and MMP family members (Table 2). Significantly up-regulated genes included the gene encoding TLR4 among the TLR family, those encoding IL-6 and IL-8 among the IL family, and the gene encoding MMP-1 among the MMP family. These data indicate that inflammatory responses to LPS are induced in NPDFs.

### LPS induces TLR4 expression in NPDFs

**Figure 2:**
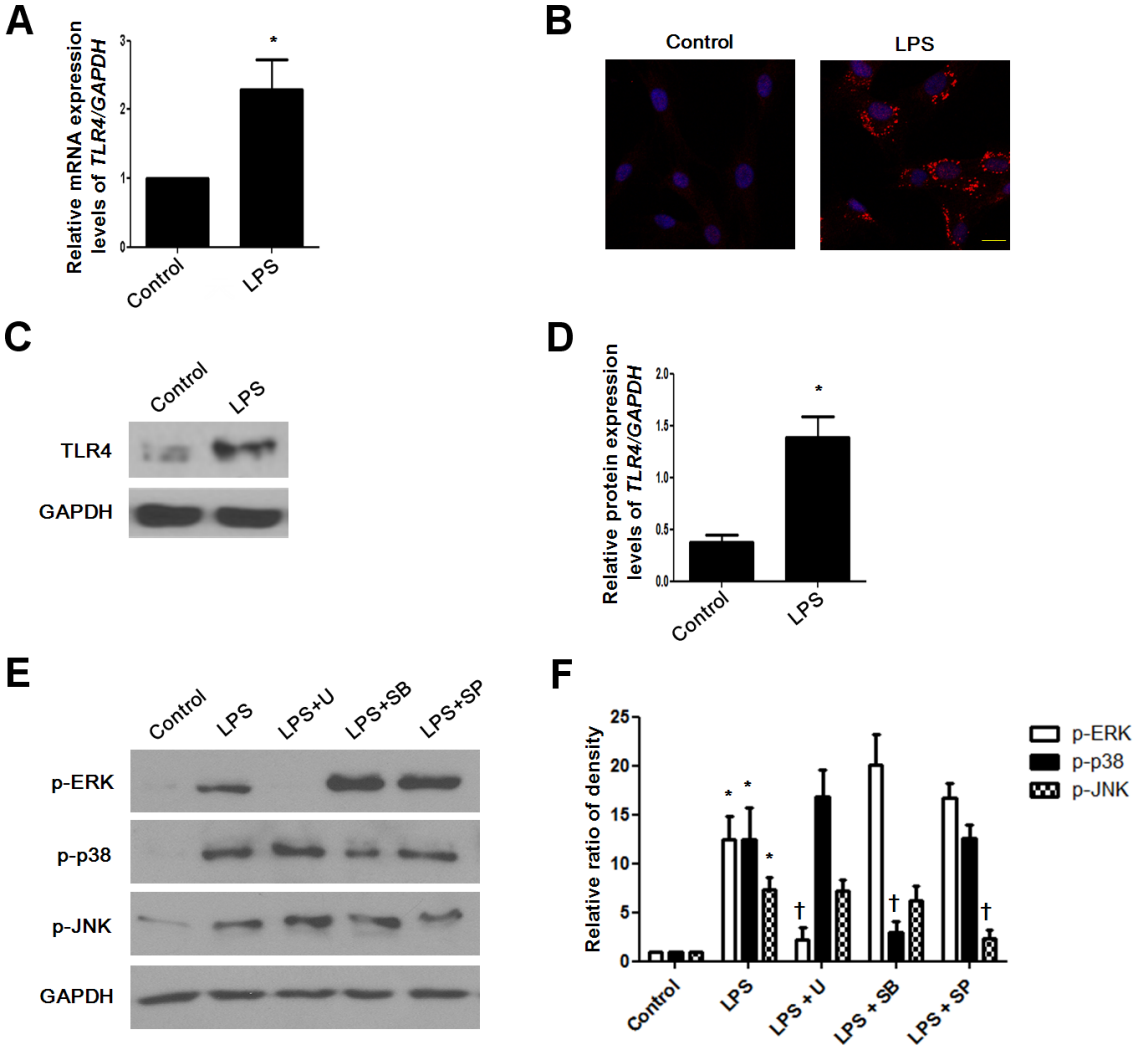
Effect of lipopolysaccharide on TLR4 expression in NPDFs. NPDFs were stimulated with LPS (10 µg/mL) in the presence or absence of each MAPK inhibitor, including U0126 (10 µM), SB203580 (10 µM), and SP600125 (10 µM). mRNA expression level of *TLR4* was determined using real-time PCR (A). TLR4 protein expression level was examined using immunocytochemical staining (B), western blotting (C) and density analysis (D). Phosphorylation of MAPK pathways involving pERK, pJNK, and p-p38 was measured using western blotting (E) and density analysis (F). Expression of GAPDH was determined as an internal control. Values are the mean ± SEM of independent samples. *Significant difference from control (* P<0.05). †Significant difference from LPS († P<0.05). Images were acquired and visualized using confocal laser scanning microscopy. Scale bar  = 20 µm.

Only *TLR4* among all *TLR* mRNA was increased in LPS-treated NPDFs (Table 2). We confirmed *TLR4* mRNA levels by using real-time RT-PCR (Fig. 2A). To identify whether LPS induces TLR4 protein expression, we treated LPS in NPDFs and evaluated expression using western blot analysis and immunofluorescence staining. The protein level of TLR4 increased in LPS-treated NPDFs (Fig. 2B, 2C and 2D). LPS phosphorylated three MAPKs, including ERK, p38, and JNK (Fig. 2E and 2F). These results suggest that LPS induces TLR4 protein expression and activates the MAPK signal pathway in NPDFs.

### LPS induces pro-inflammatory cytokine expression in NPDFs

**Figure 3:**
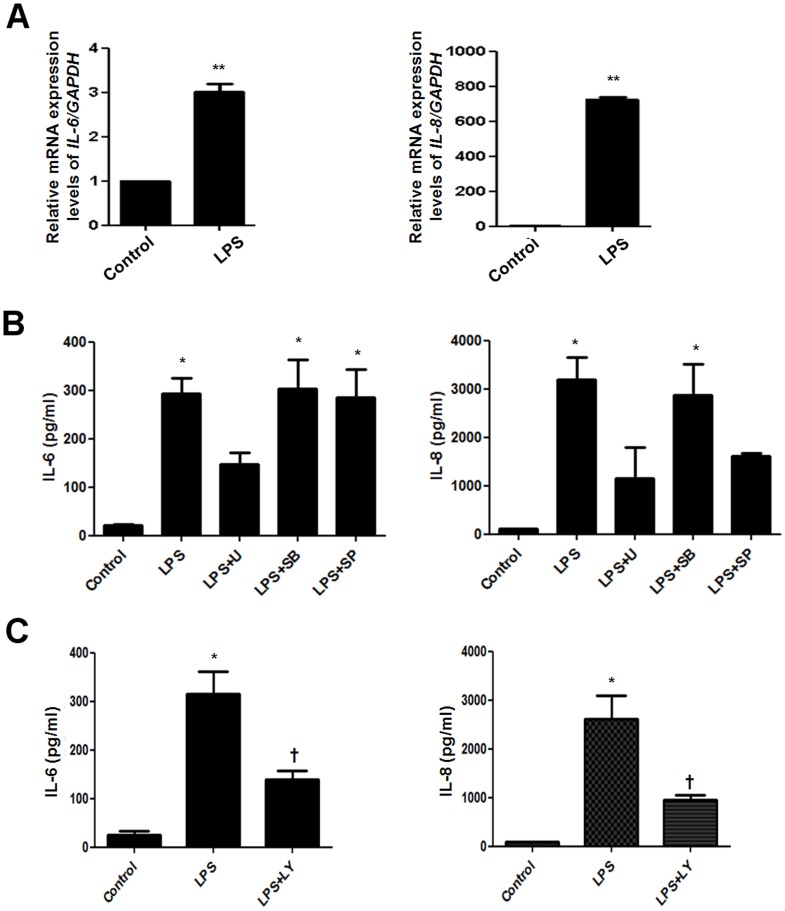
Effect of lipopolysaccharide on pro-inflammatory cytokines expression in NPDFs. NPDFs were stimulated with LPS with or without each MAPK inhibitors (U0126, SB203580, and SP600125) and PI3K/Akt inhibitor (LY294002, 10 µM). mRNA expression levels of *IL-6* and *IL-8* were examined using real-time PCR (A). (B and C) Production of IL-6 and IL-8 was measured using ELISA. Values are the mean ± SEM of independent samples. *Significant difference from control (* P<0.05 and ** P<0.01). †Significant difference from LPS († P<0.05).

To determine whether LPS induces cytokine expression in NPDFs, we stimulated fibroblasts with LPS in the presence or absence of MAPK inhibitors or PI3K/Akt inhibitor and examined mRNA and protein expression levels of various ILs. According to the microarray data, mRNA expression of *IL-6* and *IL-8* among various ILs significantly increased in LPS-treated NPDFs (Table 2). We confirmed the increased mRNA levels of *IL-6* and *IL-8* by using real-time RT-PCR (Fig. 3A). ERK inhibitor decreased IL-6 production, and ERK and JNK inhibitors decreased IL-8 production in LPS-stimulated NPDFs (Fig. 3B). Also, PI3K/Akt inhibitor decreased both IL-6 and IL-8 production in LPS-stimulated NPDFs (Fig. 3C). These results indicate that LPS induces the expression of IL-6 via ERK and PI3K/Akt pathway and the expression of IL-8 via ERK, JNK and PI3K/Akt pathway in NPDFs.

### LPS induces MMPs expression in NPDFs

**Figure 4:**
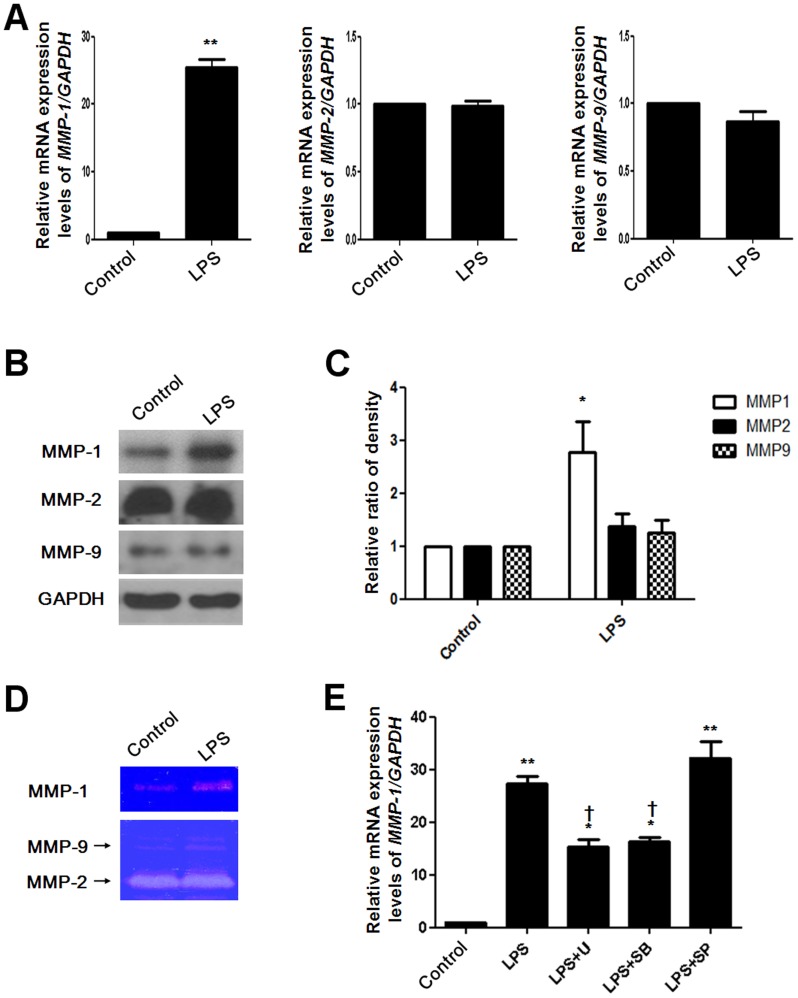
Effect of lipopolysaccharide on MMP expression in NPDFs. NPDFs were stimulated with LPS in the presence or absence of each MAPK inhibitor. Expression levels of *MMP-1*, *MMP-2*, and *MMP-9* were determined using real-time PCR (A), western blotting (B) and density analysis (C). (D) MMP-1 secretion was measured using collagen zymography. MMP-2 and MMP-9 secretion levels were measured using gelatin zymography. (E) The effect of inhibiting the MAPK pathway on *MMP-1* mRNA expression was examined using real-time PCR. Values are mean ± SEM of independent samples. * P<0.05 and ** P<0.01 compared with control; † P<0.05 compared with LPS.

A previous study showed that MMP-1 expression is increased in NPDFs [17]. Microarray data showed that *MMP-1* mRNA levels among various MMP family members significantly increased in LPS-stimulated NPDFs (Table 2). To verify the effect of LPS on MMP expression in NPDFs, NPDFs were stimulated with LPS and MMP mRNA and protein expression levels were measured. Real-time RT-PCR was used to evaluate *MMP-1*, *MMP-2*, and *MMP-9* mRNA expression levels (Fig. 4A). Protein expression levels of MMP-1, MMP-2, and MMP-9 were examined using western blot analysis (Fig. 4B and 4C). Collagenase enzyme activity of MMP-1 was measured using collagen zymography, and gelatinase enzyme activity of MMP-2 and MMP-9 was measured using gelatin zymography (Fig. 4D). MMP-1 mRNA and protein expression levels significantly increased following LPS treatment. However, MMP-2 and MMP-9 mRNA and protein levels did not increase in LPS-stimulated NPDFs. MMP-1 mRNA expression induced following LPS exposure was decreased following treatment with ERK and p38 inhibitors, but not with the JNK inhibitor (Fig. 4E). These results indicate that LPS induces not only mRNA and protein expression, but also collagenase activity of MMP-1 via the ERK and p38 signaling pathways.

### TLR4 antagonist inhibits stimulatory effects of IL-6, IL-8, and MMP1 in NPDFs

**Figure 5:**
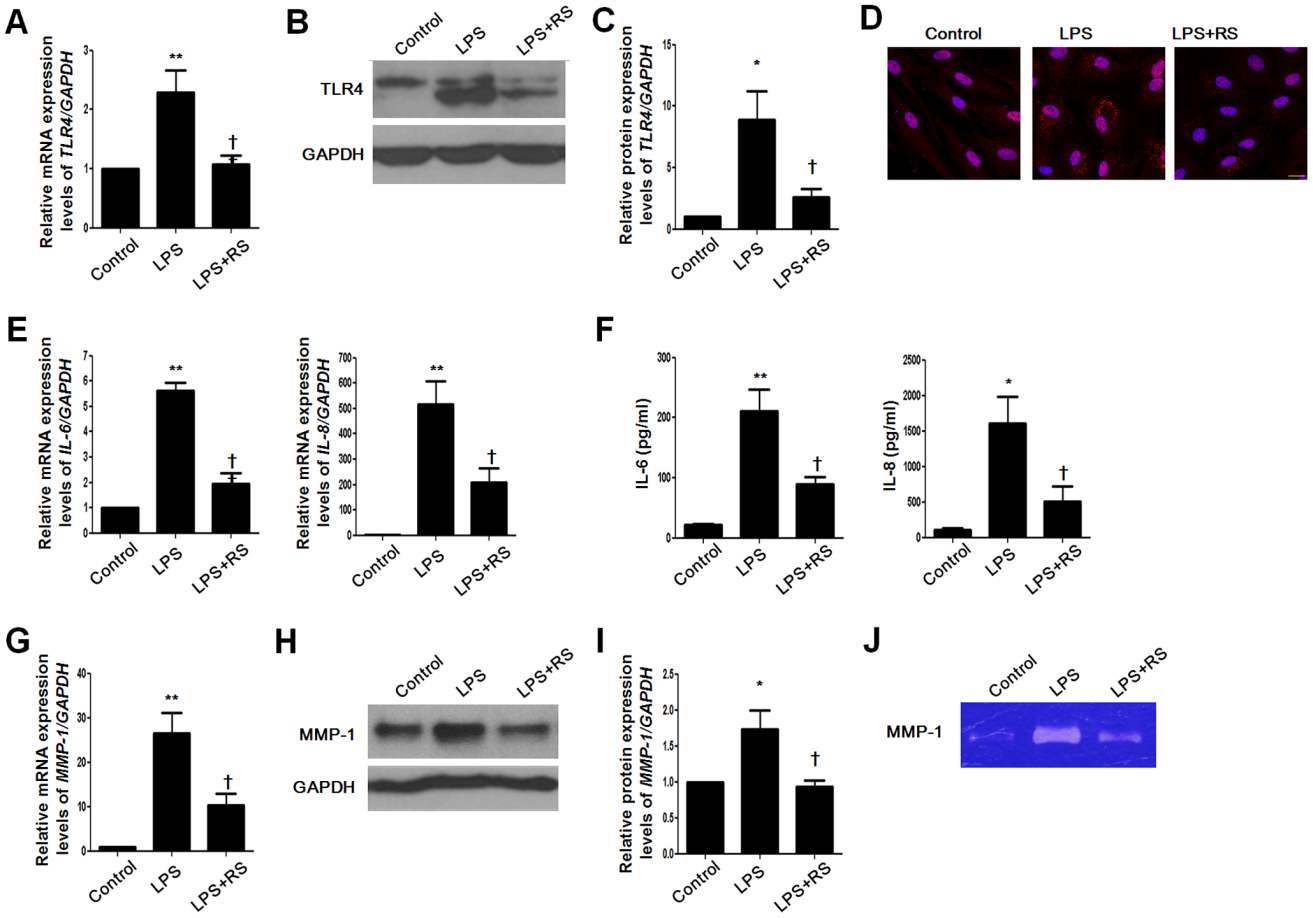
Inhibitory effect of TLR4 antagonist on pro-inflammatory cytokines and MMP-1 production in NPDFs. NPDFs were stimulated with LPS in the presence or absence of LPS-RS, a TLR4 antagonist. mRNA expression levels of *TLR4*, *IL-6*, *IL-8*, and *MMP-1* were determined using real-time PCR (A, E, and G). Protein expression levels of TLR4 and MMP-1 were examined using western blotting (B and H), density analysis (C and I) and immunocytochemical staining (D). (F) Production of IL-6 and IL-8 was measured using ELISA. (J) MMP-1 secretion was measured using collagen zymography. Values are mean ± SEM of independent samples. *Significant difference from control (* P<0.05 and ** P<0.01). †Significant difference from LPS († P<0.05 and †† P<0.01). Images were acquired and visualized using confocal laser scanning microscopy. Scale bar  = 20 µm.

LPS-RS, a potent TLR4 inhibitor, is a potent antagonist of LPS [18]. To verify the inhibitory effect of LPS-RS on the stimulatory effect of LPS, we used LPS-RS to block TLR4 function and evaluated TLR4, IL-6, IL-8, and MMP-1 expression levels. TLR4 expression was decreased after LPS-RS treatment (Fig. 5A–5C). Increased mRNA expression and protein secretion of IL-6 and IL-8 were inhibited following treatment with LPS-RS (Fig. 5D and 5F). LPS-induced MMP-1 production was blocked following treatment with the TLR4 antagonist (Fig. 5G–5J). These results imply that LPS-stimulated inflammatory responses are induced through TLR4 in NPDFs.

### LPS increases expression levels of TLR4, IL-6, IL-8, and MMP1 in nasal polyp organ cultures

**Figure 6:**
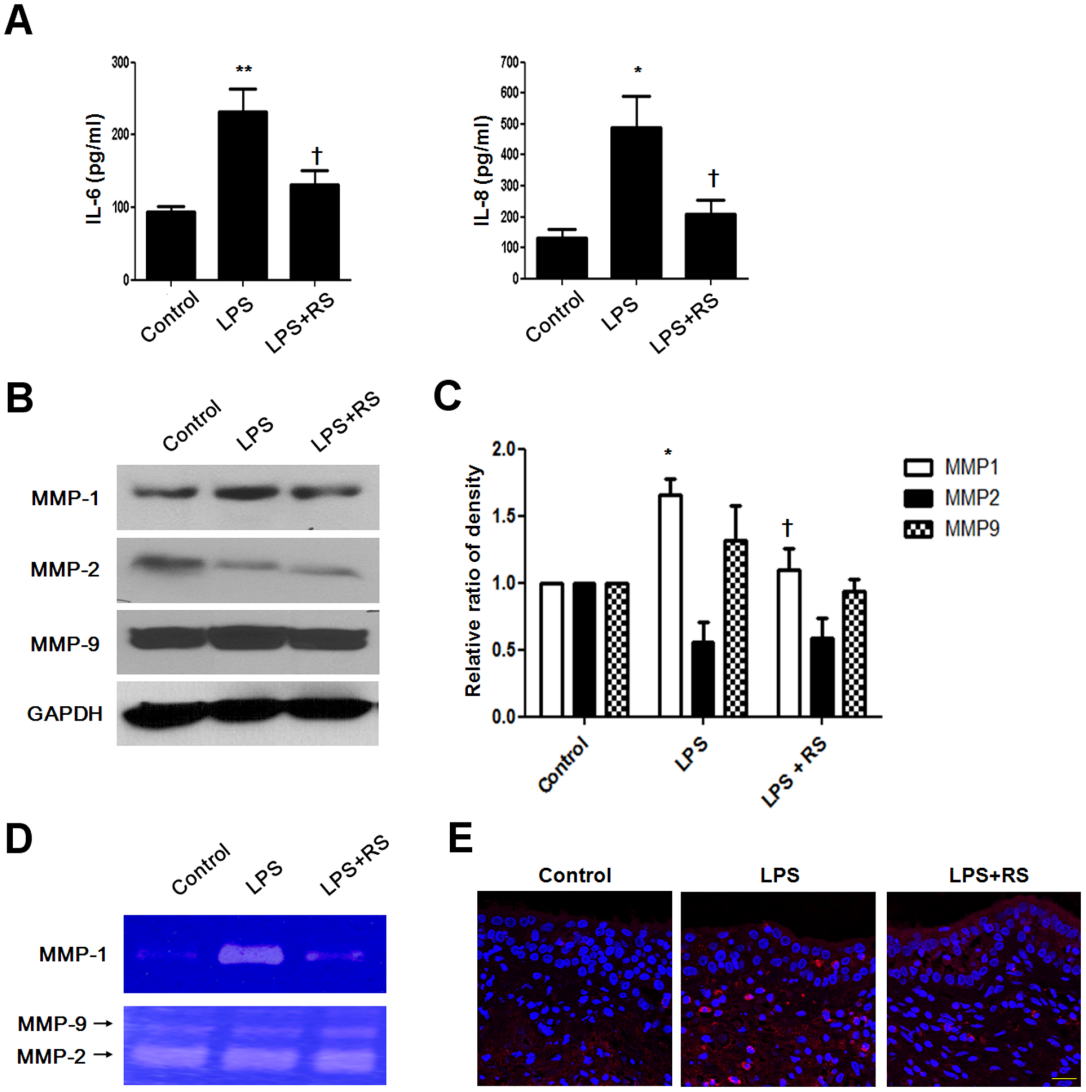
Inhibitory effect of TLR4 antagonist on pro-inflammatory cytokines and MMP-1 production in nasal polyp organ cultures. Nasal polyp tissues were cultured and stimulated with LPS with or without LPS-RS. (A) Production of IL-6 and IL-8 was measured using ELISA. Protein expression level of MMP-1 was examined using western blotting (B) and density analysis (C). (D) MMP-1 secretion was measured using collagen zymography. Secretion of MMP-2 and MMP-9 was measured using gelatin zymography. (E) The expression pattern and localization of MMP-1 by immunohistochemical staining. Values are mean ± SEM of three independent samples. Significant difference from control (* P<0.05 and ** P<0.01; † P<0.05 compared with LPS). Images were acquired and visualized using confocal z-stack laser scanning microscopy. Scale bar  = 20 µm.

Since an *in vivo* model system for nasal polyps has not been established, organ cultures of nasal polyps would be a useful research tool for investigating nasal polyp development and treatment [19], [20]. Organ cultures mimic the *in vivo* scenario because the three-dimensional structure and normal cell–cell contacts are maintained. To confirm the inhibitory effect of TLR4 antagonist on production of IL-6, IL-8, and MMP-1 in human tissues, we cultured nasal polyp organ cultures and treated the cells with LPS in the presence or absence of LPS-RS. Increased IL-6 and IL-8 production following exposure to LPS were decreased by LPS-RS in nasal polyp organ cultures (Fig. 6A). The protein expression level and collagenase enzyme activity of MMP-1 were inhibited by LPS-RS (Fig. 6B–6E). These results indicate that TLR4 antagonists inhibit LPS-induced pro-inflammatory responses in nasal polyp organ cultures.

## Discussion

The sinonasal mucosa is the first respiratory tissue encountered by environmental agents; thus, these tissues are exposed to many microorganisms as well as their breakdown products, such as LPS. These microorganisms are found in patients both with and without sinonasal diseases. An analysis of TLR expression and immune response is important for understanding the formation and remodeling of nasal polyposis. Airborne fungi stimulate TLR expression and an immune response in nasal polyp epithelial cells [21]. However, it is unknown whether LPS induces an immune response and which signal transduction pathway on immune response in nasal polyp fibroblasts. In this study, we explored the role of TLR4 in gene expression of immune responses leading to IL-6, IL-8, and MMP-1 production after LPS stimulation in nasal polyp.

MMPs involved in the degradation of ECM components; their extracellular activities are regulated by TIMP. Imbalances between MMP and TIMP activity can lead to variations in tissue remodeling. In models of experimental lung injury, levels of several MMPs were shown to be increased in bronchoalveolar lavage fluid following initiation of injury [22]. MMP-1, a member of a subfamily of matrix metalloproteinases, is known to have interstitial collagenase and fibroblast collagenase activities [23]. MMP-1 cleaves fibrillar collagen, resulting in ECM disruption. Our data confirm that MMP-1 expression is increased in nasal polyp tissues compared to inferior turbinate tissues. These results suggest that increased MMP-1 expression is associated with nasal polyp remodeling. We showed that LPS induces MMP-1 gene expression and collagenase activity and activates the ERK and p38 MAPK pathways in nasal polyp-derived fibroblasts. MMP-2 and MMP-9 increased in nasal polyps [24]. However, expressions of MMP-2 and MMP-9 are not sensitive to LPS exposure in nasal polyp fibroblasts. These results imply that LPS stimulates MMP-1 expression through the ERK and p38 signaling pathways.

Previous studies have shown that inflammatory cytokines induce MMPs, which may be a pivotal role for inflammatory cytokines such as IL-6 and IL-8 in the pathogenesis of diseases such as rheumatoid arthritis [25], chronic obstructive pulmonary disease [26], and corneal ulceration [27]. We showed that IL-6, IL-8 and MMP-1were produced by LPS in nasal polyp-derived fibroblasts. LPS derived from *E. coli* leads to the production of proinflammatory cytokines such as IL-1β, IL-6, and TNFα as well as chemokines such as IL-8 [28]. LPS derived from *Pseudomonas aeruginosa* induces IL-1α, IL-6, MCP-1, IL-1β and TNFα in airway inflammation and goblet cell hyperplasia [29]. In the previous study, LPS activated phosphorylation of the downstream mediators ERK and p38 and initiated an inflammatory response in granulosa cells through accumulation of IL-6 and IL-8 in rat synovial membranes [30]. Also, LPS induced inflammatory responses via PI3K/Akt signaling in human peripheral blood mononuclear cells [31]. In the present study, LPS activated ERK phosphorylation for IL-6 induction and induced activation of ERK and JNK, but not p38, for IL-8 induction in NPDFs. Additionally, PI3K/Akt activation increased LPS-induced IL-6 and IL-8 production in NPDFs. These data indicate that LPS stimulates IL-6 and IL-8 production through the ERK, JNK and PI3K/Akt signaling pathways in NPDFs.

LPS from the photosynthetic bacterium *Rhodobacter sphaeroides* (LPS-RS) is a potent inhibitor of TLR4 and antagonist of LPS [18]. In the present study, we found that LPS-RS inhibits the expression of LPS-stimulated TLR4, IL-6, IL-8, and MMP-1. Our data suggest that LPS induces expression of IL-6, IL-8, and MMP1 via TLR4.

A limitation of this study is that an *in vivo* model system for nasal polyposis was not established. To mimic the *in vivo* model system of nasal polyposis, we used organ cultures of nasal polyps to investigate nasal polyp development and treatment [19], [20]. The organ culture systems provide accessible tissue in an artificial environment and maintain the three-dimensional structure as well as standard cell–cell contacts. Organ cultures allow experimentation under more controlled conditions than is possible in *in vivo* experiments with a minimal alteration of natural conditions. Our study revealed that TLR4 is important for the production of pro-inflammatory cytokines and MMP-1 in nasal polyp organ cultures.

**Figure 7:**
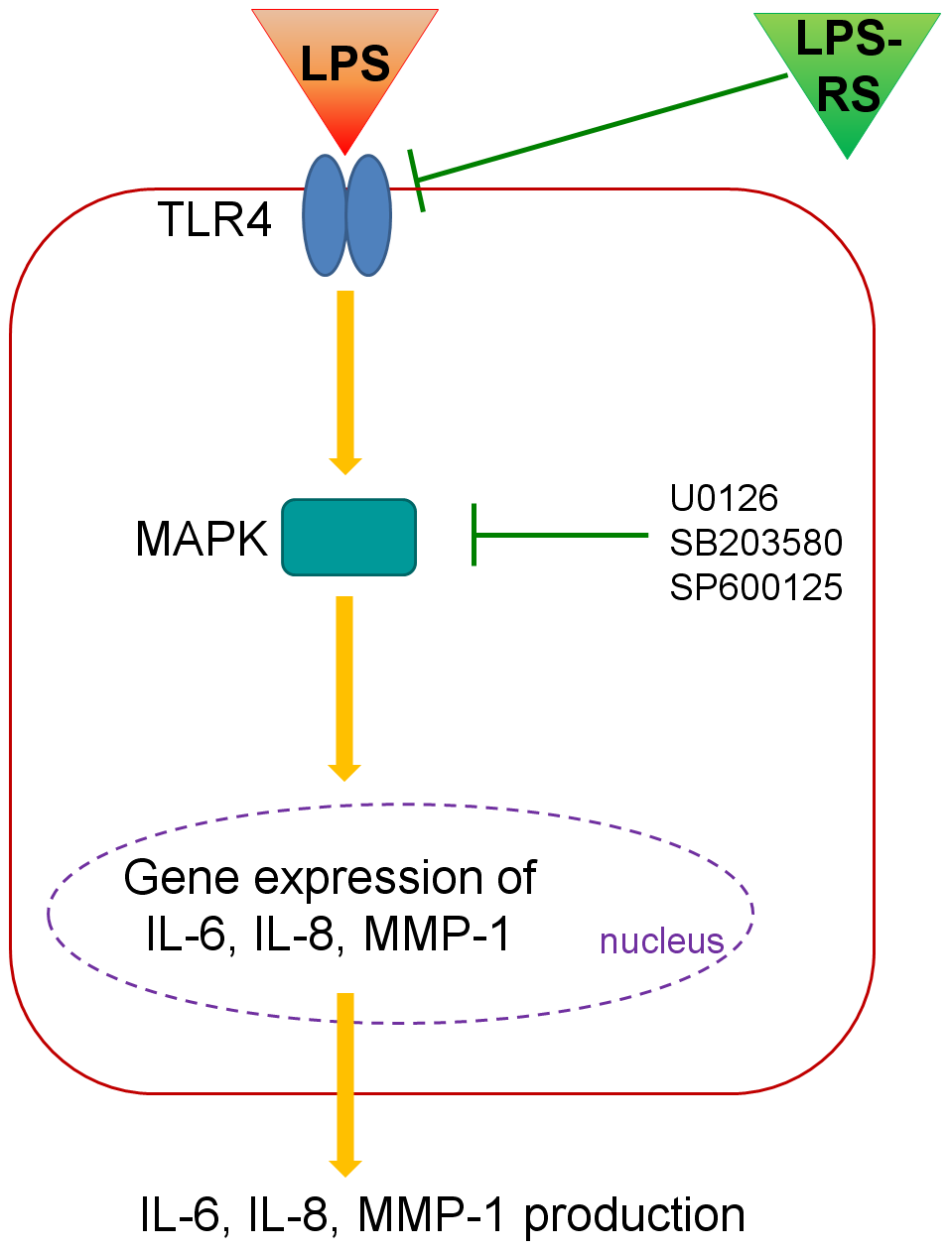
Schematic diagram for effect of lipopolysaccharide in nasal polyp-derived fibroblast and organ culture.

In summary, we showed that LPS induces production and expression of IL-6, IL-8, and MMP-1 via the TLR4, MAPK and PI3K/Akt signaling pathways in nasal polyp fibroblasts (Fig. 7). Additionally, we found that expression of IL-6, IL-8, and MMP-1 is stimulated by LPS via TLR4 in nasal polyp organ cultures. This observation indicates that LPS exposure may be involved in the pathogenesis in the remodeling of nasal polyposis.
